# Relationship between high-density lipoprotein cholesterol and the red cell distribution width in patients with coronary artery disease

**DOI:** 10.1186/s12944-018-0709-5

**Published:** 2018-03-16

**Authors:** Eyup Avci, Tuncay Kiris, Abdullah Orhan Demirtas, Hasan Kadi

**Affiliations:** 1 Cardiology Department, Balikesir University Faculty of Medicine, Balikesir, Turkey; 2 Cardiology Department, Ataturk Education and Research Hospital, Izmir, Turkey

**Keywords:** Coronary artery disease, HDL-C, Red cell distribution width, Inflammation

## Abstract

**Background:**

The red cell distribution width (RDW) is a numerical measurement of variability in the size of red blood cells. Many studies have shown that high-density lipoprotein cholesterol (HDL-C), has an anti-inflammatory effect. The aim of this study was to investigate the relationship between the serum HDL-C level and RDW in patients with coronary artery disease (CAD).

**Methods:**

Patients who underwent coronary angiography were reviewed. Patients who had moderate or severe heart failure, moderate or severe renal failure, significant systemic disease, anemia, a blood transfusion within the last 3 months, or a hematologic disease, as well as those who were taking lipid-lowering medication, were excluded from the study. The Gensini scoring system was used to determine the severity of CAD. Biochemical and hematological parameters were measured from venous blood samples taken after the patient fasted for at least 8 h. The RDW was routinely obtained from a hemogram.

**Results:**

In total, 328 patients were included in the study. The patients were categorized according to quartiles. There were 80 patients in Quartile 1 (RDW < 13.2), 84 patients in Quartile 2 (13.2 ≥ RDW < 14.15), 81 patients in Quartile 3 (14.15 ≥ RDW < 16), and 83 patients in Quartile 4 (RDW ≥ 16). There was a significant and inverse relationship between the serum HDL level and RDW. Regression analysis showed that the HDL-C, hemoglobin, and hs-CRP levels and Gensini score were predictors for the RDW.

**Conclusion:**

We found an inverse and gradual association between the serum HDL-C level and RDW, and the serum HDL-C level was an independent predictor for the RDW.

## Background

The red blood cell width (RDW), which is routinely reported in a complete blood count, is the percentage change in the size of erythrocytes [1]. An increased RDW was first detected in anemia. Later studies showed that RDW values were increased in many cardiovascular diseases, such as acute and chronic heart failure [2, 3], stroke [4], and coronary artery disease (CAD) [5, 6]. It has also been reported that an increased RDW is associated with an increased mortality and morbidity in patients with cardiovascular disease [7, 8]. It has been reported that the increase in the RDW in cardiovascular diseases may be related to increased inflammation.

Epidemiological studies have consistently shown that low levels of high-density lipoprotein cholesterol (HDL-C) are a powerful, independent predictor of CAD, that the serum HDL-C level is related to the atherosclerotic process, and that the serum HDL-C concentrations exhibited an inverse relationship with the incidence of CAD [9]. Recent studies demonstrated a powerful anti-inflammatory effect of HDL both in vivo and in vitro [10–12]. Therefore, it is reasonable to conclude that the antioxidant and anti-inflammatory properties of HDL-C may explain at least part of its anti-atherogenic potential. The aim of the present study was to investigate whether there is a relationship between HDL-C, which has anti-inflammatory properties, and the RDW, which suggests underlying inflammation in patients with CAD. Moreover, we hypothesized that there may be an inverse relationship between the serum HDL-C level and RDW.

## Methods

### Patients

Patients who underwent elective coronary angiography through the femoral artery route in the catheterization laboratory of Balikesir University Faculty of Medicine Cardiology Department were retrospectively reviewed. Patients who had a normal coronary angiogram, an acute coronary syndrome, a percutaneous coronary intervention, severe valvular heart disease, moderate or severe heart failure, coronary artery bypass grafting, renal failure, significant systemic disease, anemia, a blood transfusion within the last 3 months, or hematologic disease, as well as those who were taking lipid-lowering medication, were excluded from the study. It is known that statins have a blood HDL-C level enhancing effect. In addition, statins also have anti-inflammatory and hemorheological properties. Therefore, we did not include patients who used statins in order to create a homogeneous group. Biochemical and hematologic parameters were measured from venous blood samples taken at least 8 h after the fasting of the patient. The patients’ data were obtained from electronic records.

### High-density lipoprotein cholesterol measurements

A blood lipid profile and the serum HDL-C level were measured from after at least an 8-h fasting blood sample.

### RDW determination

The RDW was measured using an automated hematology analyzer. The patients were divided into quartiles based on their RDW values.

### Determination of the severity of CAD

The Gensini scoring system was used to determine the severity of CAD [13]. The classification system developed by Gensini is based on the degree of stenosis of the coronary lumen and localization of stenosis and includes two types of coronary angiography scores: vascular scores and stenosis scores. According to the angiographic degree of stenosis, narrowings of 0–25, 25–50, 50–75, 75–90, 90–99, and 100% were scored as 1, 2, 4, 8, 16, and 32 points, respectively. The score was then multiplied by the coefficient defined for each main coronary artery and each segment, and the results were summed. In the present study, two independent cardiologists assessed the Gensini scores.

#### Statistical analysis

The statistical software package SPSS for Windows 20.0 (SPSS Inc., Chicago, IL, USA) was used for the statistical analysis. Continuous data that fit a normal distribution are shown as the mean ± standard deviation, and those that did not fit a normal distribution are presented as the median (minimum and maximum values). Categorical variables are expressed as the frequencies and percentages. Comparisons between categorical variables were done using a chi-square test. Between-group differences in the RDW quartiles were compared using a one-way analysis of variance (ANOVA). Correlation analyses were performed using Spearman (non-normally distributed data) or Pearson (normally distributed data) correlation analyses. Logistic regression analyses were performed to determine the variables that were independently associated with the RDW. The RDW variable was categorized based on the median value (values below the median value of the RDW are encoded as “0”, and values above the median value of the RDW are encoded as “1”). In the logistic regression analysis, age, gender, body mass index, smoking, HDL-C, hypertension, hemoglobin, diabetes, hs-CRP, and Gensini score were included in the regression model as independent variables. A *p* value of less than 0.05 was considered to be significant. The study protocol was approved by the institutional ethics committee.

## Results

**Fig. 1:**
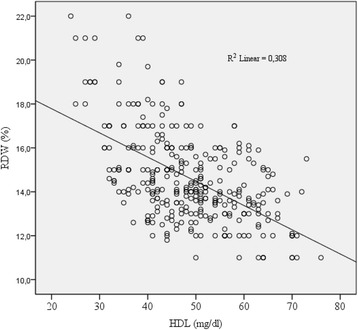
A scatterplot graphic showing the relationship between the HDL and RDW

In total, 328 patients were included in the study. When the patients were categorized according to the quartiles, there were 80 patients in Quartile 1 (RDW < 13.2), 84 patients in Quartile 2 (13.2 ≥ RDW < 14.15), 81 patients Quartile 3 (14.15 ≥ RDW < 16), and 83 patients in Quartile 4 (RDW ≥ 16).The median age (years) was 59 years in Quartile 1, 60 years in Quartile 2, 59 years in Quartile 3, and 64 years in Quartile 4. There were no significant between-group differences in age (*p* = 0.578). With regard to sex, females accounted for 44% of the patients in Quartile 1, 37% of the patients in Quartile 2, 42% of the patients in Quartile 3, and 33% of the patients in Quartile 4. The frequencies of hypertension and diabetes were similar among the RDW quartiles. The smoking status, blood creatinine, body mass index (BMI), total cholesterol, and triglyceride values were identical for the quartiles. The quartiles were comparable in terms of hemoglobin. The mean serum HDL-C levels (mg/dl) were 54.8 in Quartile 1, 51 in Quartile 2, 47.8 in Quartile 3, and 40.5 in Quartile 4. The serum HDL-C levels were significantly different between all of the quartiles. The median Gensini score was 5 in Quartile 1, 8 in Quartile 2, 10.5 in Quartile 3, and 12 in Quartile 4. The one-way ANOVA test revealed a significant difference in the Gensini scores of Quartile 1 versus Quartile 4, Quartile 2 versus Quartile 4, and Quartile 3 versus Quartile 4 (*p* < 0.0001, *p* = 0.02 and *p* = 0.019, respectively). The median hs-CRP levels (mg/dl) were 1.6 in Quartile 1, 2.3 in Quartile 2, 3.2 in Quartile 3, and 4.7 in Quartile 4. The comparison of the hs-CRP levels revealed differences between Quartiles 1 and 4 and between Quartiles 2 and 4, (*p* < 0.0001 and *p* < 0.0001, respectively). The results of the correlation analyses revealed a moderate and negative correlation between the serum HDL level and RDW (*r* = − 0.555, *p* < 0.001) (Fig. 1). The demographic, clinical, and laboratory findings of each patient are summarized in Table 1.

**Table 1:** Clinical and laboratory characteristics of the RDW quartiles

	Quartile 1	Quartile 2	Quartile 3	Quartile 4	p
Age, years,	59 (39–75)	60 (35–75)	59 (35–75)	64 (32–75)	0.578
Female, n (%)	35 (44)	31 (37)	34 (42)	27 (33)	0.237
DM, n (%)	9 (11)	11 (13)	10 (13)	12 (15)	0.587
HT, n (%)	22 (28)	35 (42)	25 (31)	29 (35)	0.639
Smoke, n (%)	33 (29)	28 (33)	24 (30)	23 (28)	0.871
hs-CRP, mg/dl	1.6 (0.3–9.4)	2.3 (0.3–9.7)	3.2 (0.5–9.3)	4.7 (0.6–9.7)	< 0.001
Hb, mg/dl	14.1 ± 0.91	14.2 ± 0.9	13.9 ± 0.94	14 ± 1	0.123
Cr, mg/dl	0.8 (0.4–1.5)	0.9 (0.5–1.5)	0.9 (05–1.5)	0.8 (0.5–1.5)	0.135
BMI, kg/m	27.5 (22–35)	27.8 (23–36)	28.8 (22–36)	28.9 (22–33)	0.732
Gensini score	5 (3–83)	8 (2–90)	10.5 (3–90)	12 (3–114)	< 0.001
HDL, mg/dl	54.8 ± 9.7	51 ± 8.6	47.8 ± 9.4	40.5 ± 8.7	< 0.001
TCHOL, mg/dl	206 ± 31	210 ± 39	198 ± 32	206 ± 38	0.221
LDL, mg/dl	124 ± 32	130 ± 34	126 ± 30	136 ± 30	0.080
TG, mg/dl	118 (36–308)	129 (49–324)	112 (45–317)	126 (42–288)	0.948

*BMI* body mass index, *Cr* creatinine, *hs-CRP* high sensitive C-reactive protein, *HDL* high-density lipoprotein cholesterol, *Hb* hemoglobin, *LDL* low-density lipoprotein cholesterol, *TG* triglyceride, *TCHOL* total cholesterol

Age, hs-CRP, Cr, BMI, TG, and Gensini score are given as median (minimum-maximum). Hemoglobin, HDL-C, TCHOL, and LDL-C are given as mean ± standard deviation

In the logistic regression analyses performed with the categorized RDW values as the accepted confounding dependent variable, it was determined that the Gensini score and hs-CRP, HDL-C, and hemoglobin levels were independent predictors for the RDW. Logistic regression analysis showed that the HDL-C, hemoglobin, and hs-CRP levels and Gensini score were predictors for the RDW. The logistic regression analysis results are shown in Table 2.

**Table 2:** Logistic regression analyses results

Variable	B	P	OR	95% CI
Age	0.09	0.511	1.009	0.982–1.036
Diabetes	−0.257	0.550	0.774	0.333–1.795
Gensini score	0.024	0.001	1.024	1.009–1.039
Gender	−0.256	0.424	0.774	0.414–1.449
Hemoglobin	−0.514	0.001	0.598	0.439–0.815
HDL-C	−0.100	< 0.000	0.904	0.876–0.934
hs-CRP	0.108	0.048	1.300	1.007–1.817
BMI	0.064	0.187	1.066	0.969–1.173
Smoking	0.432	0.136	1.539	0.873–2.714
Hypertension	−0.418	0.158	0.659	0.369–1.177

*HDL-C* high density lipoprotein cholesterol, *hs-CRP* high sensitivity C-reactive protein, *BMI* body mass index

## Discussion

The present study revealed an inverse and gradual association between the blood HDL-C level and RDW in patients with CAD and additionally an inverse correlation between the serum HDL-C and hs-CRP levels. To the best of our knowledge, this is the first study to evaluate the relationship between the RDW and serum HDL-C level in patients with CAD.

Coronary artery disease is a leading cause of death and disability worldwide. Despite scientific advances in the area, the exact mechanism underlying the pathophysiology of atherosclerosis remains unclear. In the majority of cases, the underlying cause of CAD is atherosclerosis, which is a chronic inflammatory disease of an unknown cause [14]. Firstly, Constantinides [15] showed that plaques in the coronary artery were characterized by marked inflammation and macrophage infiltration and that inflammation was a leading cause of CAD. Consistent with the literature, we found a close correlation between the Gensini score and CRP level in the ANOVA analyses. In the correlation analysis, there was a weak but significant correlation between the CRP level and Gensini score (Table 3).

**Table 3:** Correlation analyses results

	r	p
HDL-RDW	−0.555	< 0.001
Gensini score- CRP	0.204	< 0.001
HDL- GENSİNİ score	−0.193	< 0001
RDW-CRP	0.356	< 0.001

*HDL* high density lipoprotein cholesterol, *RDW* red blood cell distribution, *CRP* C-reactive protein

The relationship between the serum HDL-C level and atherosclerosis has been known for a long time. Reduced levels of HDL-C are associated with an increased risk for coronary artery disease and future cardiovascular events [16]. At least some of these anti-atherogenic effects of HDL-C may be due to its anti-inflammatory and antioxidant properties, which shows that the anti-inflammatory and antioxidant properties of HDL-C remain unquestionable in previous studies [17–20]. We found a significant correlation between the serum HDL-C level and Gensini score, which indicates the anti-atherogenic properties of HDL (Table 3).

There is evidence that high RDW values are associated with chronic inflammation and oxidative stress [21]. It has been reported that an increased RDW in various cardiac diseases, such as heart failure and coronary artery disease, reflects the underlying inflammatory state [22–24]. In a study by Lippi et al. that included a large-scale and unselected patient population, higher RDW levels were found to be associated with lower HDL-C levels, and they reported that an elevated RDW is associated with underlying low-grade inflammation [25]. Because our study was conducted in a selected group of patients (in patients with coronary artery disease), it differs from that of Lippi. However, the results of our study confirm the results of the aforementioned study. In the present study, we found a gradual and significant relationship between the CRP level and RDW quartiles in the ANOVA analyses, and HDL-C was an independent predictor for the RDW. In another study, Gang and colleagues [26] reported that those with high RDW values during a 4-year follow-up were at a greater risk for DM development. Researchers have speculated that an increased RDW may be associated with inflammation insomuch that it has been shown that the lowering of cholesterol by statins leads to a decrease in the RDW [27]. Given that atherosclerosis is an inflammatory disease, a lower Gensini score and lower CRP levels in the low RDW quartile may indicate a lower inflammatory activity in patients in this group. In addition, we showed a moderate and significant correlation between the CRP level and RDW (Table 3). Findings from previous studies and the present study suggest that an increased RDW may be a consequence of increased inflammatory activity in patients with coronary artery disease. In both the ANOVA and correlation analyses, we found a significant and inverse correlation between the serum HDL-C level and RDW. The correlation coefficient between the serum HDL-C and CRP levels was very high (Table 3). Moreover, the serum HDL-C level was an independent predictor for the RDW. These results of our study can be explained by the anti-inflammatory properties of HDL-C, and the present study once again highlights the anti-inflammatory properties of HDL-C.

Nutrition has a very significant effect on blood lipid levels. While malnutrition has a negative effect on blood lipid levels, there are studies in which compounds called nutraceuticals have positive effects on blood lipid levels and that show that they are effective in slowing atherosclerosis and protecting cardiovascular diseases in primary and secondary (antioxidant and anti-inflammatory) effects. However, in a meta-analysis of 104 studies, Scicchitano and colleagues [28] concluded that the positive effects of nutraceuticals on blood lipid parameters and the cardiovascular system were doubtful. In our study, we did not investigate the dietary characteristics of patients. The patients in our study group were from the same geographical area and were diagnosed for the first time, and none of the patients stated that they had a special diet. Therefore, the effect of nutrition can be considered to be the same for all of the patients in this study.

### Study limitations

First, this is a retrospective study and the study population was limited. Second, the objective markers of inflammation have not been studied. Another limitation of our study is that it lacks a control group. We believe that more comprehensive studies will be useful for clarifying the anti-inflammatory properties of HDL-C.

## Conclusion

The present study revealed an inverse and gradual association between the blood HDL-C level and RDW in patients with CAD. Furthermore, the serum HDL-C level was an independent predictor for the RDW. It might be speculated that the results of the present study can be explained by the anti-inflammatory properties of HDL-C.

## Abbreviations

CAD: Coronary artery disease
HDL-C: High-density lipoprotein cholesterol
RDW: Red cell distribution width
